# Parallel analysis of finite element model controlled trial and retrospective case control study on percutaneous internal fixation for vertical sacral fractures

**DOI:** 10.1186/1471-2474-14-217

**Published:** 2013-07-23

**Authors:** Hongwei Chen, Lijun Wu, Rongmei Zheng, Yan Liu, Yang Li, Zihai Ding

**Affiliations:** 1 Department of Orthopedics, Yiwu Central Hospital, Wenzhou Medical College, Yiwu 322000, China; 2 Wenzhou Medical College, Institute of Digitized Medicine, Wenzhou, Zhejiang 325035, China; 3 Anatomical Institute of Minimally Invasive Surgery, Southern Medical University, Guangzhou 510515, China

**Keywords:** Vertical sacral fractures, Percutaneous internal fixation, Biomechanical stability, Biomechanical compatibility, Finite element model controlled trial, Clinical case study

## Abstract

**Background:**

Although percutaneous posterior-ring tension-band metallic plate and percutaneous iliosacral screws are used to fix unstable posterior pelvic ring fractures, the biomechanical stability and compatibility of both internal fixation techniques for the treatment of Denis I, II and III type vertical sacral fractures remain unclear.

**Methods:**

Using CT and MR images of the second generation of Chinese Digitized Human “male No. 23”, two groups of finite element models were developed for Denis I, II and III type vertical sacral fractures with ipsilateral superior and inferior pubic ramus fractures treated with either a percutaneous metallic plate or a percutaneous screw. Accordingly, two groups of clinical cases that were fixed using the above-mentioned two internal fixation techniques were retrospectively evaluated to compare postoperative effect and function. Parallel analysis was performed with a finite element model controlled trial and a case control study.

**Results:**

The difference of the postoperative Majeed standards and outcome rates between two case groups was no statistically significant (P > 0.05). Accordingly, the high values of the maximum displacements/stresses of the plate-fixation model group approximated those of the screw-fixation model group. However, further simulation of Denis I, II and III type fractures in each group of models found that the biomechanics of the plate-fixation models became increasingly stable and compatible, whereas the biomechanics of the screw-fixation models maintained tiny fluctuations. When treating Denis III fractures, the biomechanical effects of the pelvic ring of the plate-fixation model were better than the screw-fixation model.

**Conclusions:**

Percutaneous plate and screw fixations are both appropriate for the treatment of Denis I and II type vertical sacral fractures; whereas percutaneous plate fixation appears be superior to percutaneous screw fixation for Denis III type vertical sacral fracture. Biomechanical evidence of finite element evaluations combined with clinical evidence will contribute to our ability to distinguish between indications that require plate or screw fixation for vertical sacral fractures.

## Background

**Figure 1:**
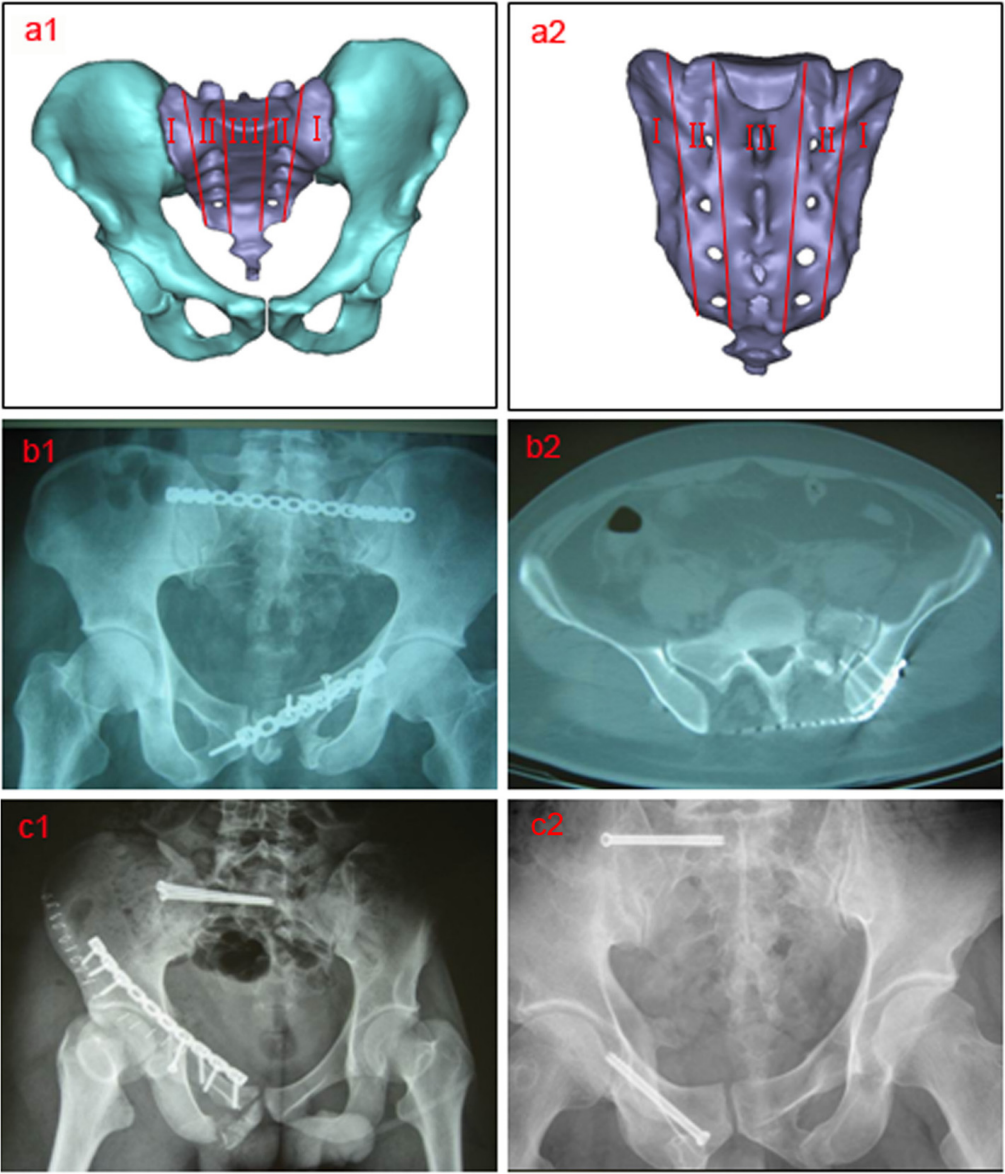
**Sacral fracture typing by Denis. (a)** Denis I, II and III zones of the sacrum (a1 anterior view, a2 posterior view); **(b)** a case (male, age 38) of a Denis II type left sacral fracture treated with percutaneous PTMP (b1 anteroposterior X-ray, b2 transverse plane CT scan); **(c)** a case (male, age 25) of a Denis II type right sacral fracture and a case (male, age 36) of Denis I type right sacral fracture both treated with percutaneous SIJS (anteroposterior X-ray).

The sacrum is a mechanical nucleus that serves as the base for the spinal column as well as the keystone of the pelvic ring. Thus, injuries of the sacrum can lead to both biomechanical instability and nerve conduction abnormality [1,2]. Sacral fractures can be classified using the Denis classification into three types: type I is an ala region fracture with an incidence of approximately 50%; type II is a foramina region fracture with an incidence of approximately 34%; and type III is a central sacral canal fracture with an incidence of approximately 16% [1,3] (Figure 1(a)). Vertical displaced sacral fractures (DSFs) usually result from high-energy traumas [2,4], and are often associated with sacroiliac joint (SI joint) dislocations and pelvic anterior-ring fractures, which then are classified as completely unstable (Type C) according to the Tile classification for pelvic fractures; their reported mortality rate can be as high as 10% [5]. The treatment of vertical sacral fractures may result in complications, such as fracture malunion, post-traumatic nonunion, delayed sacral nerve injury and late-onset low back pain. At present, many experts advocate the reduction of fracture and reconstruction of the three-dimensional stability of the anterior- and posterior-ring, as well as for its ability to diminish the likelihood of late complications [5-8].

The posterior-ring tension-band metallic plate (PTMP) and sacroiliac joint screw (SIJS) are two commonly used methods for posterior internal fixation of the pelvis [9]. With the development of a minimally invasive surgical technique, percutaneous PTMP and percutaneous SIJS have been increasingly performed in clinical scenarios (Figure 1(b) and 1(c)), thus diminishing pelvic surgical trauma, shortening surgical time, and reducing the rate of perioperative complications [10-12]. However, as when treating different types of vertical sacral fractures, the biomechanical stability and compatibility of these two percutaneous techniques remain unclear. In this study, using the concepts of evidence-based medicine, we conducted a parallel analysis of biomechanical finite element (FE) model controlled trial and retrospective clinical case study on percutaneous PTMP and SIJS fixations for three types of vertical sacral fractures (Denis I, II and III types). The hypothesis for the present study was that percutaneous PTMP and SIJS fixations may both appropriate for the treatment of Denis I and II type vertical sacral fractures; whereas percutaneous PTMP fixation may be superior to percutaneous SIJS fixation for Denis III type vertical sacral fractures.

## Methods

This study was approved by the Medical Ethics Committee of Wenzhou Medical College of China.

### FE modeling of the intact pelvis of the second generation Chinese digitized human

On Mimics 11.0 (Materialise Company, Belgium) and Ansys 11.0 (Ansys Company, USA) software platform, a FE model of an intact bony pelvis was developed from the 3D reconstruction of 165 CT images with 1.25-mm slice thickness and increment of the second generation Chinese Digitized Human (F2-CDH) “male No. 23” (the digitized model of a Chinese volunteer with standard body figure, male, 23 years old, height 169 cm, weight 65 kg) [13]. The element types of the bony medium containing cortical and cancellous bones, the matrix of a SI joint capsular ligament (SIJCL), the cartilage of SI joint, and the cartilage of acetabulum, interpubic disc, etc., were defined as 3D solid elements. Meanwhile, according to MRI information of F2-CDH, the ligamentous tissue FE models were attached to the bony model. The element types of the ligamentous tissue including SIJCL fibers, anterior sacroiliac ligaments (ASIL), posterior sacroiliac ligaments (PSIL), interosseous sacroiliac ligaments (ISIL), sacrospinous ligaments (SSL), sacrotuberous ligaments (STL), superior pubic ligaments (SPL), arcuate pubic ligaments (APL), pectineal ligaments (PL) and inguinal ligaments (IL), were determined to the 3D cable elements.

A contact model of cartilages of the SI joint was established by a slidable plane to plane contact elements with a gap of 0.1 mm and a friction coefficient of 0–0.48 [14,15]. The matrix of the SIJCL was defined as the hyperelastic material in line with the Mooney-Rivlin 2-parameter law. Its elastic modulus ranged from 2.146 Mpa to 4.291 MPa, and the Poisson ratio as 0.49 [14,16]. The SI joint model was classified into three types: SI1 (normal state), SI2 (slight weaken state), SI3 (moderate weaken state). The effects of three SI joints on pelvis biomechanical stability were quite similar, then, the relatively unfavorable and unstable SI joint model (SI3) was used for FE analysis. The material properties of different components of the pelvis are listed in Table 1[14-21]. The numbers of elements, nodes and contact planes of the intact pelvis of F2-CDH are shown in Table 2.

**Table 1:** **The material properties of a series of FE models of intact and fixed pelvises**[14-21]

**Item**	**Elastic modulus**	**Poisson ratio**	**Frictional coefficient**	**Cross section area**
	***E *(MPa)**	***μ***	***f***	***A *(mm**^**2**^**)**
Titanium plate	110000	0.30	0.45	–
Titanium screw	110000	0.30	–	–
Cortical bone	17000	0.3	0.4	–
Cancellous bone	129	0.2	0.4	–
Articular cartilage	11.9.40-0.48	–		
Interpubic disc	5	0.45	–	–
SIJCL matrix	2.146-4.291	0.49	–	–
SIJCL fibers	105	0.3	–	392.00
Pelvic ligaments				
ASIL	251.3	0.3	–	19.38
PSIL	251.3	0.3	–	79.40
ISIL	251.3	0.3	–	66.24
SSL	251.3	0.3	–	32.22
STL	251.3	0.3	–	79.74
SPL	251.3	0.3	–	12.82
APL	251.3	0.3	–	7.43
PL	251.3	0.3	–	6.76
IL	251.3	0.3	–	20.26

Note: SIJCL matrix (Mooney-Rivlinhyperelasticity): W = C_10_(I_1_-3) + C_01_(I_2_-3), (C_10_ + C_01_) = E/4(1 + *μ*), *E* = 2.146–4.291 MPa, *μ* = 0.49; I_1_ and I_2_ are the first and second invariants of the Cauchy-Green tensor. The first type of SI joint (SI1) simulates to approach normal state (*E*_1_ = 4.291 MPa; *μ*_1_ = 0.49; *f*_1_ = 0); the second type of SI joint (SI2) simulates slight weaken state (*E*_2_ = 3.128 MPa; *μ*_2_ = 0.49; *f*_2_ = 0.24); the third type of SI joint (SI3) simulates moderate weaken state (*E*_2_ = 2.146 MPa; *μ*_2_ = 0.49; *f*_2_ = 0.48) [14,21].

**Table 2:** The number of elements, nodes, and contact planes in a series of FE models of intact and fixed pelvises

**Model**	**Solid element**	**Link element**	**Objective element**	**Contact element**	**Total element**	**Total node**	**Sum contact surface**
Intact	82408	130	273	430	83241	22127	2
P1	101147	123	1039	1261	103570	26497	8
P2	102643	123	1053	1264	105083	27259	12
P3	102309	123	1101	1307	104840	27403	10
S1	94587	123	700	943	96353	25093	6
S2	95543	123	724	963	97353	25525	10
S3	95968	123	794	1012	97897	25517	8

### FE modeling of vertically fractured pelvis fixed with percutaneous PTMP and SIJS

According to the digitized pelvic model of F2-CDH and the radiological images of clinical cases with vertically sacral fractures (Figure 1(a), 1(b), 1(c)), as well as the percutaneous technique of pelvic surgery, the internal fixation models of percutaneous PTMP and percutaneous SIJS in the fractured posterior pelvic ring were constructed in detail: (i) the PTMP is fixed between the bilateral posterior superior iliac spines and the superior border of the first sacral foramen, and three screws are used to lock the bilateral ilia, respectively. The screws penetrate the bilateral SI joints at the sacral cortex, without penetrating the fractured surfaces of the sacrum (Figure 1(b)); (ii) the insertion site of the percutaneous SIJS lies in the posterior superior iliac spine, and the cannula is parallel to the superior border of the first sacral foramen. Under CT guidance, the pin penetrates the fracture surfaces, but needs to avoid the sacral canal and the sacral nerve foramen (Figure 1(c)); (iii) when constructing the anterior pelvic ring disruption model of ipsilateral superior and inferior pubic ramus fractures, the prebendingmoulding metallic plate (PMMP) (or a screw) is fixed on the superior ramus of the fractured pubis, which should cross over the fracture surface but not always stride across the interpubic disc (Figure 1(b) and 1(c)) [11,12].

The interface model of screws and surrounding bone tissue is regarded as the multi-medium continuum using shared nodes to simulate firm internal fixations. The relationship between the metallic plate of the anterior/posterior ring and surrounding bony tissue was described as a slidable plane to plane contact elements with a friction coefficient of 0.45. Accordingly, the fracture surface models of the sacrum as well as the superior and inferior pubic rami were defined by a slidable plane to plane contact elements with a gap of 0.1 mm and a friction coefficient of 0.4 [22]. According to the most unfavorable principle, the ligament injury models of anterior-posterior pelvic ring fractures were defined as follows: Denis I type sacral fracture accompanied partial injuries of the PSIL, Denis II and III type sacral fractures accompanied partial injuries of the PSIL, SSL and STL, the superior and inferior pubic rami fractures resulted in injuries of the PL [6,14].

**Figure 2:**
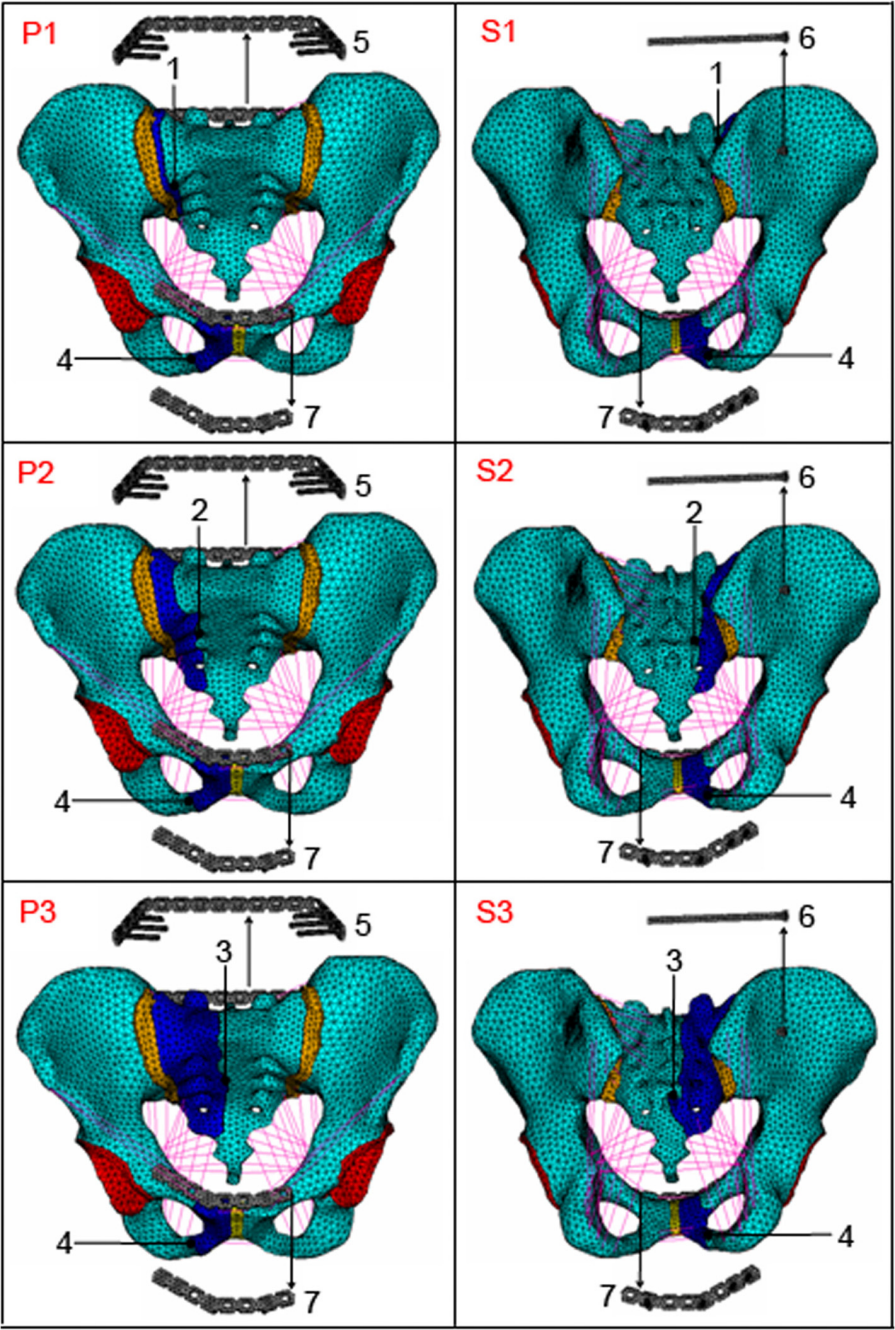
**(P1), (P2), (P3) A group of FE models of vertical sacral fractures (Denis I, II and III types) with ipsilateral superior and inferior pubis rami fractures treated with percutaneous PTMP, respectively (anterior view).** (S1), (S2), (S3) Another group of FE models of vertical sacral fractures (Denis I, II and III types) with ipsilateral superior and inferior pubis rami fractures treated with percutaneous SIJS, respectively (posterior view). 1 The sacral fracture surface of a Denis I type fracture; 2 the sacral fracture surface of a Denis II type fracture; 3 the sacral fracture surface of a Denis III type fracture; 4 fracture surface of superior and inferior pubis rami; 5 percutaneous PTMP; 6 percutaneous SIJS; 7 anterior-ring PMMP.

Finally, two groups of finite element models were developed for Denis I, II and III type vertical sacral fractures with ipsilateral superior and inferior pubic ramus fractures treated with percutaneous PTMP in the posterior-ring and PMMP in the anterior-ring (P1, P2, P3) vs. percutaneous SIJS in the posterior-ring and PMMP in the anterior-ring (S1, S2, S3), as shown in Figure 2. The material properties of titanium plate and titanium screw are listed in Table 1. The numbers of elements, nodes and contact planes in the two groups of surgical FE models of pelvises are shown in Table 2.

### Localization validation of intact and surgical FE models of the pelvises

When the intact and surgical FE models of the pelvises simulating balanced standing phase, the constraints of the models were located on bilateral acetabular fossa cartilage (with an average thickness of 1.46 mm [19]) and bilateral acetabular lips of fibrocartilage (with an average thickness of 2.0 mm [20]), all of which were fixed in three-direction translation components. The upper weight loads of 500 N were added on the superior surface of the sacrum (or base of sacrum) (sharing 85% of the load) and bilateral superior articular facets (sharing 15% of the load) of the first sacral vertebra on the basis of the spinal three-column theory [16,21].

The intact FE model of the pelvis was validated as follows: (i) under the same experimental conditions as that of the balanced standing pelvis, the model-predicted peak vertical displacements (1.239 mm to 1.758 mm under 500 N vertical loads) were coincident with the corresponding experiment-measured peak compressive displacements (0.973 to 1.550 mm under 500 N vertical loads) reported by Wu et al. [23] and Comstock et al. [24]; (ii) the FE simulations indicated that the posterior-ring contributed approximately 61.5%-69.5% to the stiffness of intact pelvis. The computational values were in agreement with the clinically reported values (60%~70%) published by Tile [6] and Chen et al. [12]. (iii) under similar experimental conditions of the hip bones positioned upside down as reported by Dalstra et al. [18], the FE model-predicted peak von Mises stresses (4.838-8.333 MPa under 500 N loads) of a hip-bone material and femoral-head load sensitivity analysis agreed with the experiment-found peak von Mises stress (6.625 MPa under 600 N loads).

The surgical FE model of the pelvis (S1, Denis I type) was validated as follows. When the posterior-ring was fixed but the anterior-ring unfixed, the calculations of FE models of SIJS-fixation found that the vertical displacements were approximately 1.174~1.609 mm under 500 N vertical loads, which were close to the experimental results of approximately 1.69 mm under the same fixation modes and load conditions measured by Comstock et al. [24], who utilized solo SIJS to treat unilateral SI joint dislocation (its injury surface close to Denis I type fracture surface) with ipsilateral superior and inferior pubis ramus fractures.

### Parallel analysis for two groups of surgical FE models and two groups of clinical cases

A FE model controlled trial [25,26] was performed to compare the biomechanical differences between two groups of surgical models (P-fixation group vs. S-fixation group) in the treatment of Denis I, II and III type fractures. The computation loads and boundary conditions of two groups of surgical FE models referred to the balance-standing phase of the intact pelvis model. The compressive state implemented a 500 N vertical load; the flexion state implemented a 500 N vertical load and a 10 Nm moment of forward sagittal direction; the lateral bending state implemented a 500 N vertical load and a 10 Nm moment of right lateral direction. Within two groups of surgical models, two displacement indexes were defined as the maximum sum displacement of the pelvic ring ($U_{\max}^{R}$) and the maximum vertical displacement of the injured sacrum ($Z_{\max}^{S}$), meanwhile two stress indexes were defined as the maximum von Mises stress of internal fixator ($\sigma_{\max}^{F}$) and the maximum von Mises stress of bony tissue of pelvic ring ($\sigma_{\max}^{B}$), which were used to represent the postoperative biomechanical stability and compatibility of the pelvic ring [16,21,23,24].

A retrospective study of two groups of clinical cases was investigated to parallel two groups of surgical FE models [25,26]. Thirty-three patients with vertical sacral fractures (including Denis I, II and III types) and ipsilateral superior and inferior pubic rami fractures were selected between March 2002 and October 2007 [12]. They were divided into two groups according to two kinds of internal fixations, percutaneous PTMP fixation (P group, n = 17 cases) and percutaneous SIJS fixation (S group, n = 16 cases). All cases underwent closed fixations of the anterior and posterior rings (Figure 1(b) and 1(c)). The general states of the two groups of cases are listed in Table 3, and their age, sex, sacral fracture type, and follow-up time (1 to 3 years) were all comparable (*P* > 0.05). Postoperative X-ray and CT scans were performed to evaluate the fracture reductions and plate/screw positions. The postoperative complications in two groups were recorded. Data of the postoperative Majeed standards, and outcome rates, were recorded and analyzed statistically in SPSS 15.0 (SPSS Company, USA). Majeed function assessment included pain (30 points), work (20 points), sitting (10 points), sexual intercourse (4 points) and standing (36 points) [27]. Clinical grade was determined as: excellent ≥85 points, 85 points > good ≥70 points, 70 points > fair >55 points, poor ≤55 points [27]. A two-sample *t*-test was used for the Majeed standards, while the ordinal polytomous logistic regression was applied for the excellent and good rates. A *P*-value <0.05 was defined as the level of statistical significance.

**Table 3:** The general states of two groups of clinical cases

**Index**	**P group (n = 17)**	**S group (n = 16)**	***P-*value**
Age (year)	35.6 ± 9.9	39.8 ± 11.4	0.277
Sex (male:female)	13:4	11:5	0.619
Sacral fracture type (I:II:III)	5:10:2	5:11:0	0.364
Follow-up time (month)	25.5 ± 5.7	23.0 ± 5.6	0.217

Note: The statistics for age and follow-up time used a two-sample *t*-test; the statistics for sex used a chi-square test with a four-fold table; the statistics for sacral fracture type used a chi-square test with a R × C table.

## Results

### Biomechanical comparison of the pelvic ring between two groups of surgical FE models

**Figure 3:**
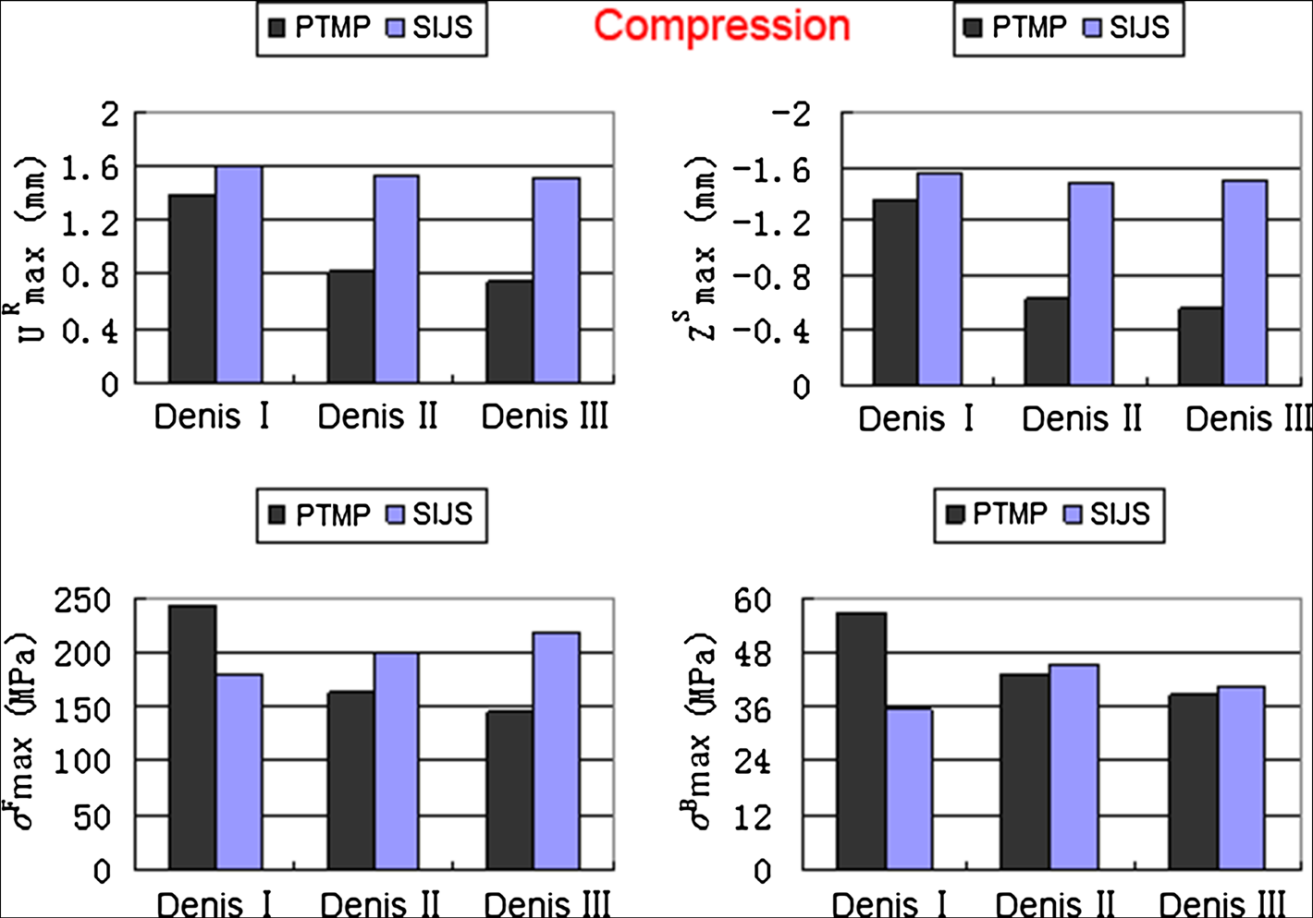
**The maximum sum displacement of the pelvic ring (**$U_{\max}^{R}$**) and the maximum vertical displacement of the injured sacrum (**$Z_{\max}^{S}$**), the maximum von Mises stress of internal fixator (**$\sigma_{\max}^{F}$**) and the maximum von Mises stress of bony tissue of pelvic ring (**$\sigma_{\max}^{B}$**) of two groups of surgical FE models (PTMP vs. SIJS) under compression states.**

**Figure 4:**
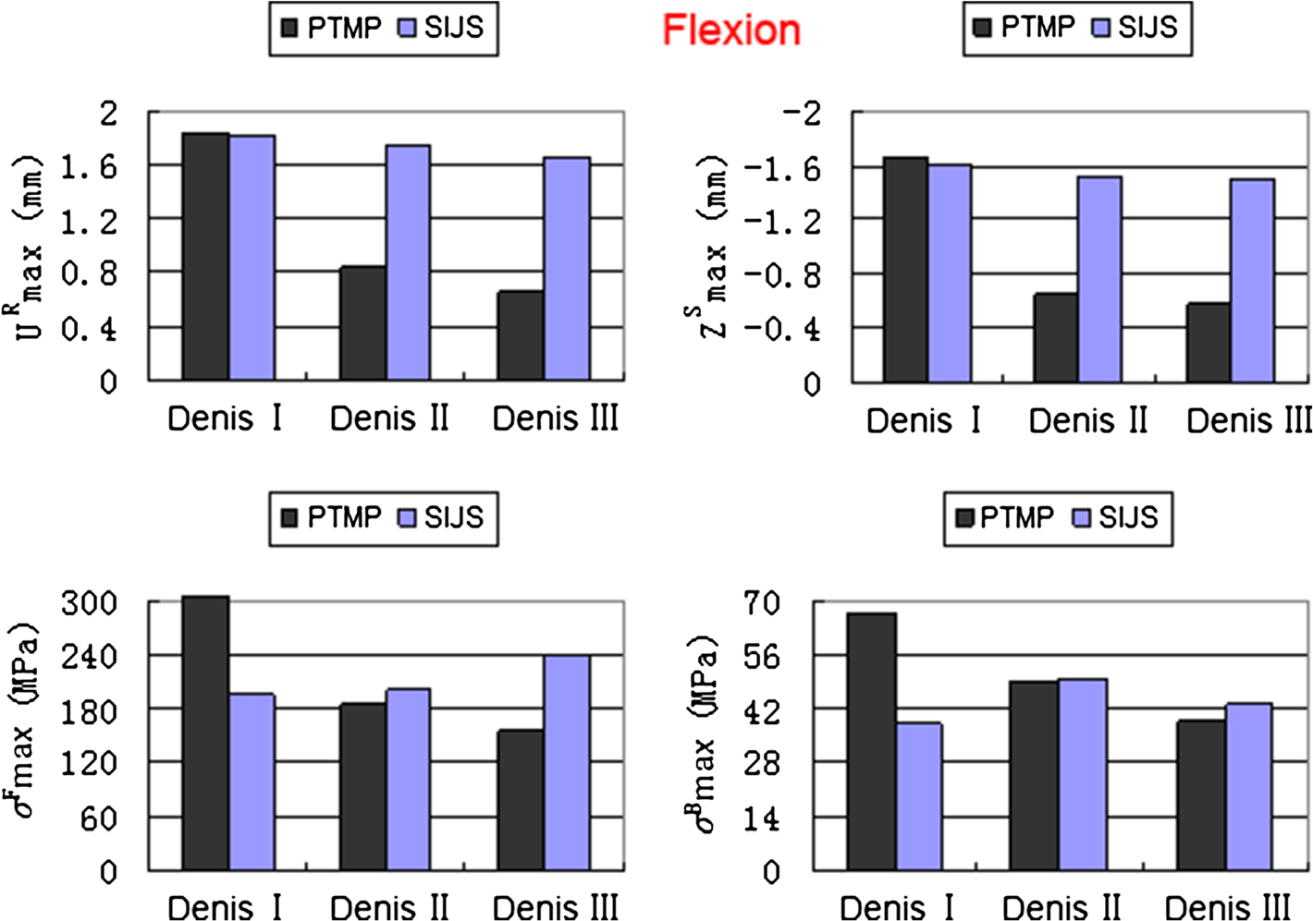
**The maximum sum displacement of the pelvic ring (**$U_{\max}^{R}$**) and the maximum vertical displacement of the injured sacrum (**$Z_{\max}^{S}$**), the maximum von Mises stress of internal fixator (**$\sigma_{\max}^{F}$**) and the maximum von Mises stress of bony tissue of pelvic ring (**$\sigma_{\max}^{B}$**) of two groups of surgical FE models (PTMP vs. SIJS) under flexion states.**

**Figure 5:**
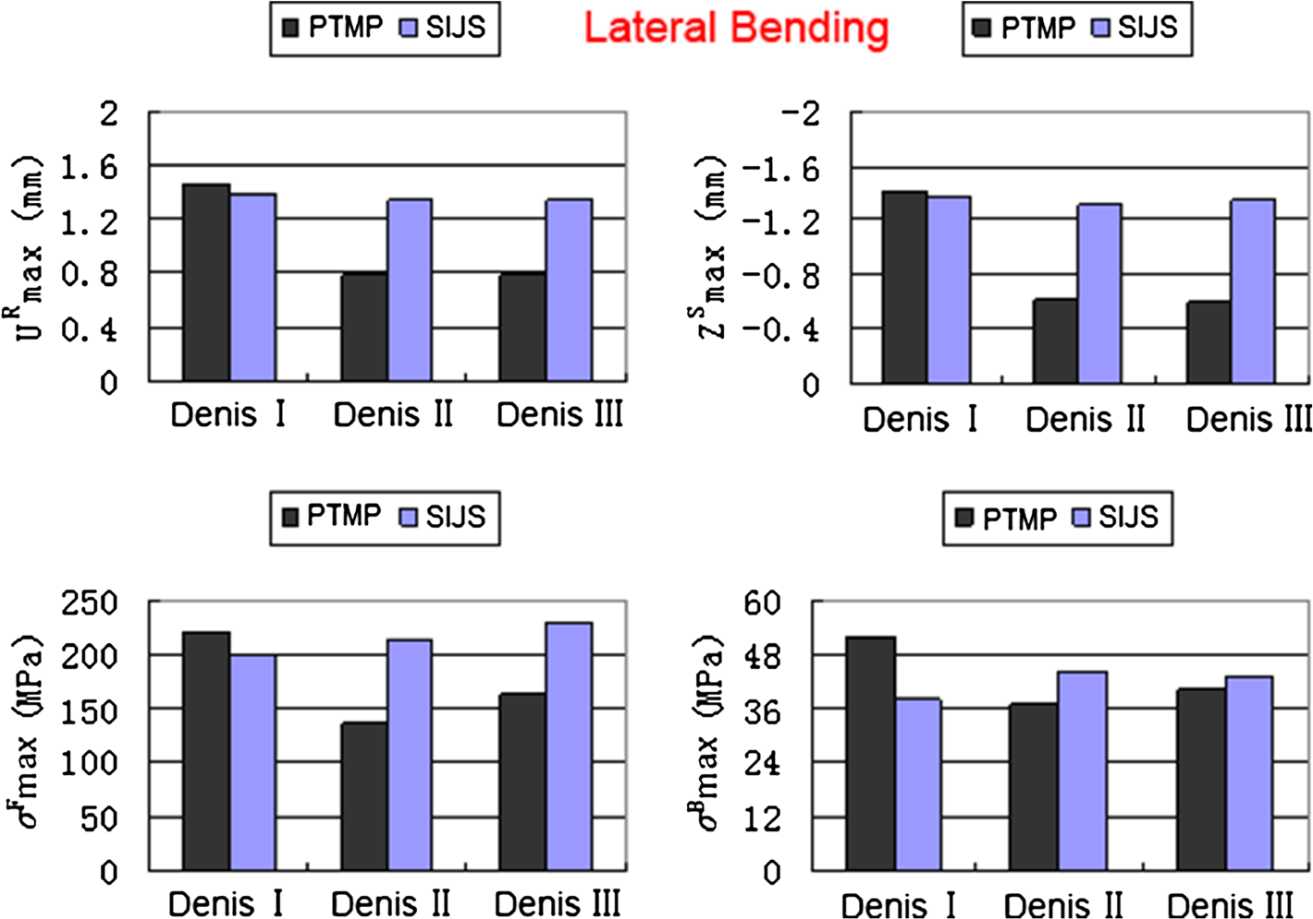
**The maximum sum displacement of the pelvic ring (**$U_{\max}^{R}$**) and the maximum vertical displacement of the injured sacrum (**$Z_{\max}^{S}$**), the maximum von Mises stress of internal fixator (**$\sigma_{\max}^{F}$**) and the maximum von Mises stress of bony tissue of pelvic ring (**$\sigma_{\max}^{B}$**) of two groups of surgical FE models (PTMP vs. SIJS) under lateral bending states.**

Under the compression, flexion and lateral bending states, the maximum displacements and the maximum von Mises stresses of two groups of surgical FE models (including Denis I, II and III type fractures) all occurred in their posterior pelvic rings. The high values of the maximum displacements/stresses of the plate-fixation model group approximated those of the screw-fixation model group. However, the low values of the maximum displacements/stresses the plate-fixation model group were obviously less than those of the screw-fixation model group, as shown in detail in Figures 3, 4 and 5, respectively.

When simulating between Denis I, II and III type sacral fractures of P-fixation models, the $U_{\max}^{R}$ decreased obviously with an average value of 52.03%, $Z_{\max}^{S}$ also decreased markedly with an average value of 60.24%; Meanwhile, $\sigma_{\max}^{F}$ reduced markedly with an average value of 38.68%, and $\sigma_{\max}^{B}$ also reduced significantly with an average value of 32.66%. All indexes indicated that the biomechanical stability and compatibility effects of percutaneous PTMP fixation models improved in Denis III type sacral fractures. However, when further simulating between Denis I, II and III type sacral fractures of S-fixation models, the displacement indexes ($U_{\max}^{R}$ and $Z_{\max}^{S}$) showed a slight decrease with average values of 6.16% and 4.14%, respectively, but the stress indexes ($\sigma_{\max}^{F}$ and $\sigma_{\max}^{B}$) displayed a slight increase with average values 18.95% and 13.84%, respectively. This demonstrated the biomechanical stability and compatibility of percutaneous SIJS fixation models might maintain a tiny fluctuation.

When treating Denis III type sacral fractures under compression states, the displacement indexes ($U_{\max}^{R}$, $Z_{\max}^{S}$) and the stress index ($\sigma_{\max}^{F}$, $\sigma_{\max}^{B}$) of plate-fixation models were diminished by 50.43%, 62.67%, 33.30% and 5.44% compared to those of screw-fixation models, respectively. Under flexion states, the $U_{\max}^{R}$, $Z_{\max}^{S}$, $\sigma_{\max}^{F}$ and $\sigma_{\max}^{B}$ of plate-fixation models were diminished by 59.98%, 61.49%, 34.99% and 9.11%, respectively compared to those of screw-fixation models. Under lateral bending states, the $U_{\max}^{R}$, $Z_{\max}^{S}$, $\sigma_{\max}^{F}$ and $\sigma_{\max}^{B}$ of plate-fixation models were also diminished by 41.62%, 55.06%, 29.12% and 7.65% compared to those of screw-fixation models, respectively. This indicated that biomechanical stability and compatibility of pelvic rings of percutaneous PTMP fixation models were, in general, better than percutaneous SIJS fixation models for the treatment of Denis III type sacral factures.

**Figure 6:**
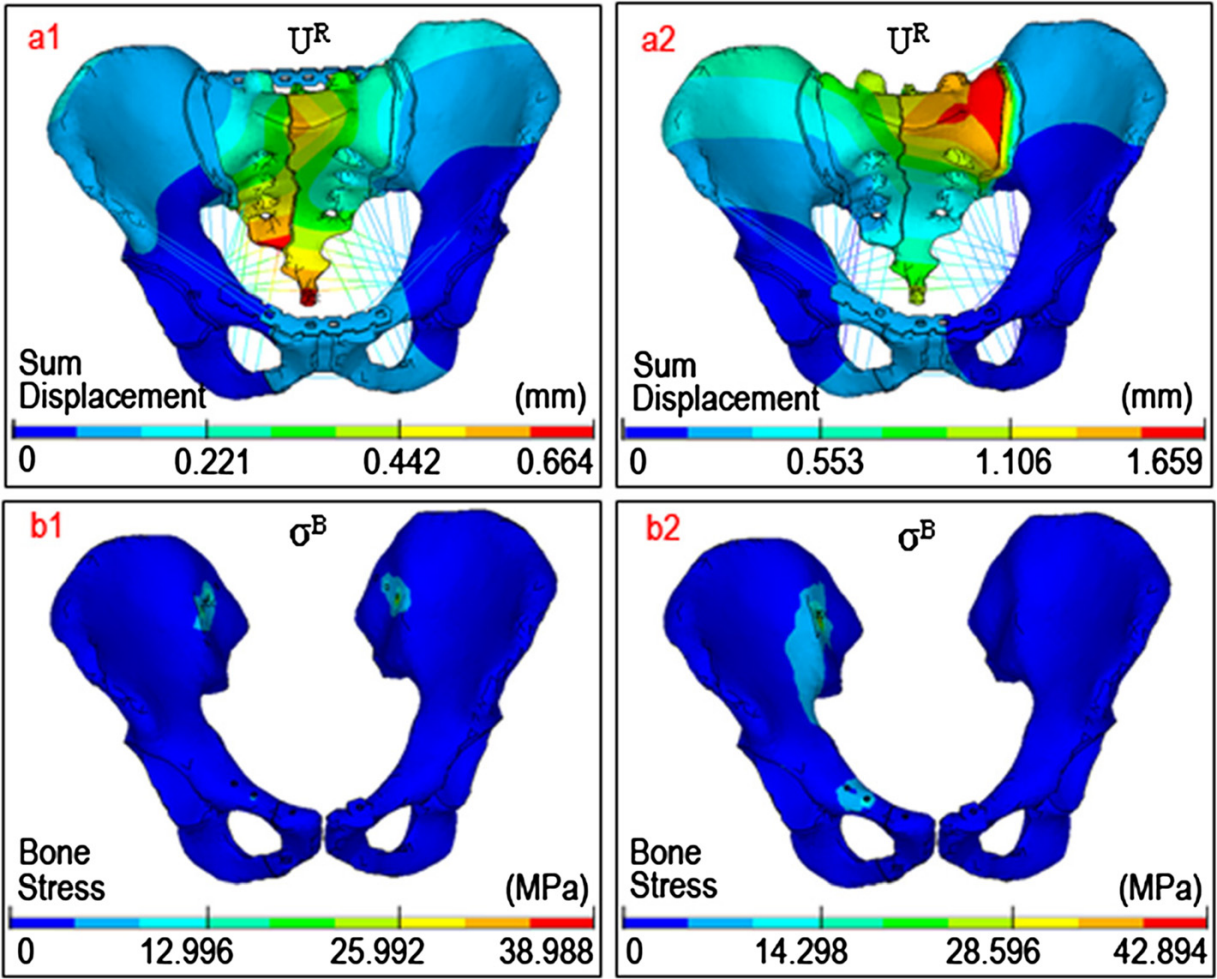
**The distribution nephogram of displacements/stresses.** The representative distributions of **(a)** the sum displacements and **(b)** von Mises stresses in two surgical FE models with Denis III type fractures (PTMP vs. SIJS) both under flexion states.

Finally, in fractured sacral zones I, II or III, the displacement distributions and the stress distributions in the posterior pelvic ring of the P-fixation models all showed progressive symmetry, whereas the S-fixation models had no symmetry. Moreover, the peak von Mises stresses of the sacral fracture surfaces of P-fixation models were obviously lower than those of S-fixation models. The most obvious symmetry emerged at sacrum zone III fractures of the P-fixation models, as shown in Figure 6.

### Comparison of clinical efficacy between the two groups of surgical cases

**Figure 7:**
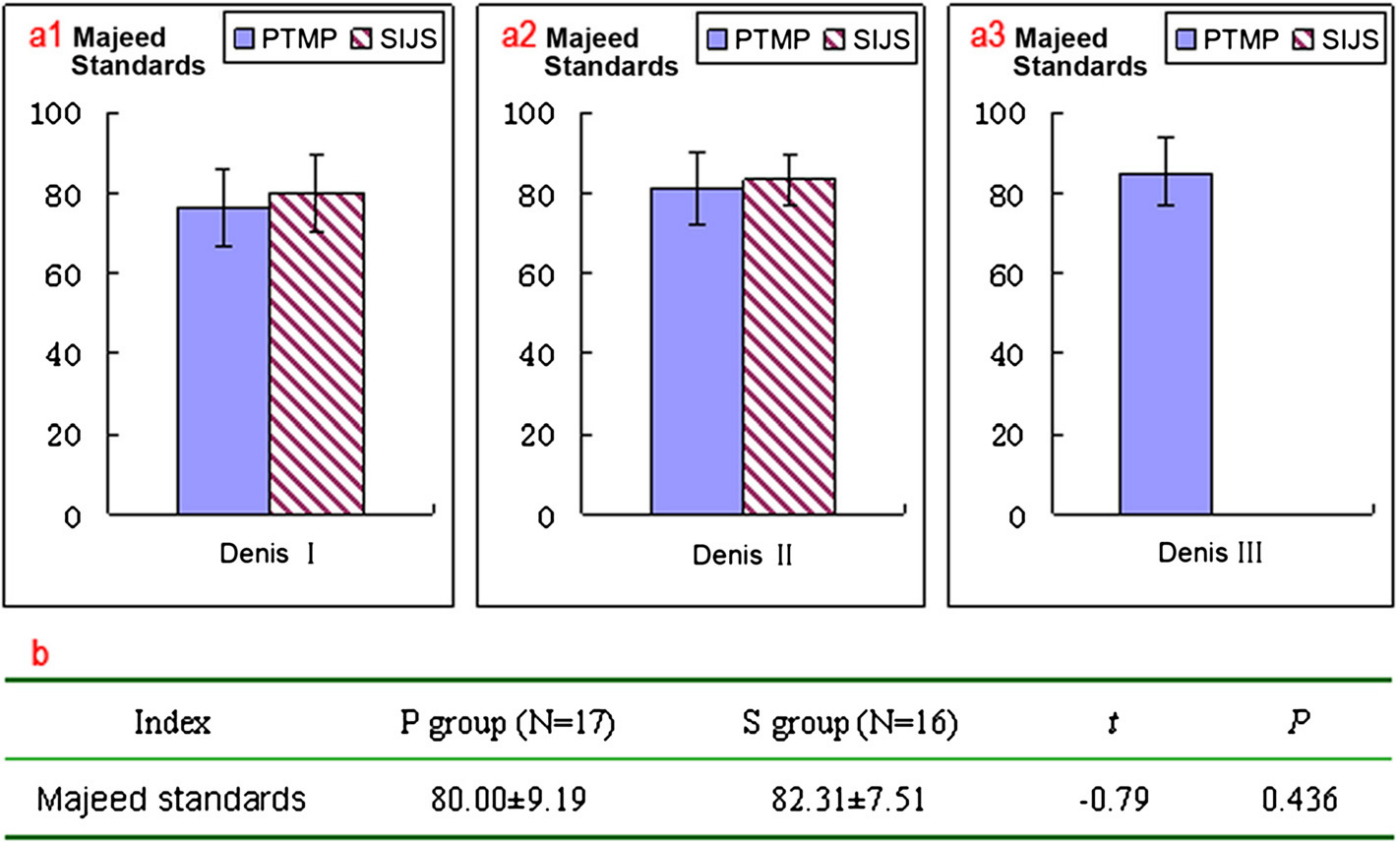
**The function assessments of the case groups. (a)** Postoperative Majeed standards of two groups of clinical cases (percutaneous PTMP vs. percutaneous SIJS) with different types of vertical sacral fractures, respectively. **(b)** Statistical analysis of postoperative Majeed standards between two groups of clinical cases (percutaneous PTMP vs. percutaneous SIJS).

Postoperative X-ray and CT scans demonstrated that both groups of patients achieved satisfactory reductions, and the PTMP and SIJS were fixed in satisfactory positions (Figure 1(b) and 1(c)). All 33 patients in two groups had no damage to blood vessels during the operation, showed no infection at the incision, no loosening or disruption of the internal fixation after operation, while their fractures all healed. However, in one patient of S group, the screw was fixed in the sacral foramina which injured the sacral nerve. The symptoms improved after screw replacement in revision surgery and drug use for the nutrient nerve. In two patients of S group, the screws were too short, but did not extract from the sacrum. Postoperative Majeed standards of the P group cases were between 62 and 93 points, with an average of 80.0 points. Within the total, there were six excellent cases, nine good cases, and two fair cases. The rate of excellent and good outcomes was 88.2%. Postoperative Majeed standards of the S group cases ranged from 71 to 94 points, with an average of 82.3 points. Within the total, there were six excellent cases, 10 good cases, and no fair cases. The excellent and good rate was therefore 100%. When percutaneous PTMP fixation cases including Denis I, II, III type sacral fractures and percutaneous SIJS fixation cases including Denis I, II type sacral fractures were compared, the postoperative Majeed standards, and the excellent and good rates of the two groups of clinical cases, did not demonstrate any statistically significant differences (*P* > 0.05), as can be seen in Figure 7.

## Discussion

The major surgical targets of percutaneous PTMP and SIJS treatment for vertical sacral fractures of posterior pelvic ring are to reduce the pelvis maximally, to stabilize the fracture effectively, to reconstruct lumbosacral spine alignment, and to promote the recovery of nerve function [4,11,28]. The postoperative biomechanics of the posterior pelvic ring play a key role for the realization of surgical targets of both percutaneous internal fixation techniques. The present FE models indicated that the major loads of the pelvic ring were shared and burdened by the posterior-ring, which contributed approximately 61.5%-69.5% to the stiffness of the pelvis. The stress indexes and displacement indexes of pelvic ring reflected the biomechanical compatibility and biomechanical stability of the pelvic internal fixations. According to the principle of stress concentration and stress shielding [14], increasing postoperative pelvic stress approximates its natural stress, as well as the smaller the stress difference between the pelvis and the internal fixator becomes, the better the biomechanical compatibility that can be achieved by an internal fixation system [16,21]. Meanwhile, the smaller the postoperative displacement of pelvic ring becomes, the better the biomechanical stability that will be obtained by an internal fixation [23,24].

The controlled trial of two groups of surgical FE models interestingly indicated that, when simulating between Denis I, II and III type sacral fractures, the maximum displacement indexes and maximum stress indexes of P-fixation models obviously reduced by approximately 52.03%-60.24% and 32.66%-38.68%; thus, their pelvic ring biomechanics were increasingly robust and compatible, whereas the biomechanical effects of the screw-fixation models maintained tiny fluctuations. Considering a vertically unstable sacral fracture type such as the Denis III type, the maximum displacement indexes and maximum stress indexes of plate-fixation models were significantly decreased by 41.62%-62.67% and 5.44%-34.99%, respectively compared to the screw-fixation models under the same physiological loads; this showed the mechanical stability of the plate-fixation model was stronger and more robust than that of the screw-fixation model, and the risk of fatigue injury of the plate fixation system was less than that of screw-fixation system. Furthermore, the mechanical compatibility between the pelvis and plate fixation system was better than that between the pelvis and the screw fixation system. It was thus clear that among Denis III type sacral factures, the biomechanical effects of percutaneous PTMP fixation models were general superior to those of the percutaneous SIJS fixation models.

The present retrospective case control study, as well as the clinical report of Chen et al. [12] demonstrated that, although both internal fixations of percutaneous PTMP and SIJS achieved satisfactory curative effects, the cases fixed with percutaneous PTMP had no associated compression of the sacral foramen and canal, no injuries to the sacral nerves or the pelvic great vessels, and had a lower requirement for an intraoperative radiological perspective; therefore, percutaneous PTMP fixation can be used to treat all three types of vertical sacral fractures (case numbers of I, II and III fracture types were 5, 10 and 2, respectively). However, the percutaneous SIJS fixations were used to treat Denis I and II type sacral fractures, but might be high risky and dangerous to fix Denis III type sacral factures [12] (case numbers of I, II and III fracture types were 5, 11 and 0, respectively). Matta and Saucedo [7] used SIJS to treat sacroiliac joint dislocation or disruption, and their study demonstrated that SIJS fixation was most consistent with the biomechanical principle of centricity fixation. However, percutaneous SIJS fixation might encounter difficulty in placing the screws, and may risk damage to the sacral nerves, caudaequina and adjacent blood vessels [11,12,29]. Chen et al. indicated that Denis type III sacral fractures may be a surgical contraindication for SIJS fixation owing to the inadequate length of the screw and the high risks of screw placements [12].

Accordingly, the FE models predicted that as the PTMP was fixed in the bilateral posterior superior iliac spines and the bilateral sacral cortex, the compressive stress of the fracture surface of the PTMP fixation models was obviously less than that of the SIJS fixation models. When percutaneous PTMP was used to treat Denis III type sacral fractures, the stress distributions and the displacement distributions represented an anatomical and biomechanical rationale for the symmetrical fixation of the pelvic posterior-ring tension band, and its biomechanical effects proved to be better than the centricity fixations of the percutaneous SIJS. This mechanism was similar to the closed pelvic inter-lock system, and was favored for its stabilization and union of the vertical sacral fractures close to the central sacral canal.

The retrospective study of a small and rather inhomogeneous clinical case series is potentially underpowered for supporting the indication of sacral fracture internal fixation. Fortunately, a biomechanical FE model controlled trial in parallel with retrospective case control study can make up the lack of clinical evidence, and it is in line with the principle of evidence-based medicine [25,26]. Denis III type sacral fractures were not observed in clinical case group of the SIJS fixations which might be high risky and dangerous [12], and a risky operation usually caused safety problems and ethical conflicts. However, the FE model trial could well interpret the biomechanical difference between the PTMP fixation and SIJS fixation for Denis III fractures, and this work could be realized in a virtual experiment platform without any operation risks [13].

The present FE models of percutaneous PTMP and SIJS fixations still contain certain approximations and limitations. Firstly, our FE modeling adopted the experimental technique in vitro irrespective of the effects of pelvic muscles and fascia on pelvic stability, as well as the effects of pelvic bony density and elastic modulus on internal fixation strength, and may therefore differ from pelvic biomechanics in vivo [13,14]. However, we believe that both surgical FE models of percutaneous PTMP and SIJS fixations are simulated in the same experimental conditions in vitro and same bony material properties, which can reliably distinguish between the mechanical differences of these two internal fixations. Secondly, two groups of surgical FE models were developed with one metallic plate or one SI screw fixed in posterior-ring, as well as both with one plate fixed in anterior-ring, that could identify their biomechanical difference under the comparable operation condition. Clinically, Denis II type sacral fractures may use two SI screws to increase its stability and security; sometimes a plate of anterior-ring may be replaced with one screw which need not penetrate through the interpubic disc [12]. Thirdly, the use of the Denis classification was also a limitation of our surgical FE models (as it is a classification describing sacral fracture localizations and solely gives information on the frequency of concomitant neurological injuries [3,29]). Actually, the surface of vertical sacral fractures and the injury degree of pelvic ligaments are complex, which may complicate sacroiliac joint dislocation, as well as bilateral or multi-direction sacral fractures [6,8]. Therefore, with regards to the relatively complex posterior pelvic ring unstable fractures, the FE models of internal fixation require further design improvements with the use of biomechanical and evidence-based clinical research.

## Conclusions

In clinic, percutaneous posterior-ring tension-band metallic plate and percutaneous iliosacral screws are both appropriate for the treatment of Denis I and II type vertical sacral fractures. In the FE model used, percutaneous plate fixation is superior to percutaneous screw fixation for the treatment of Denis III type vertical sacral fractures. The biomechanical evidence of finite element evaluations combined with clinical evidence will distinguish between the indications for plate or screw fixation in vertically unstable posterior pelvic fractures.

## Abbreviations

DSF: Displaced sacral fractures; SI: Sacroiliac joint; PTMP: The posterior-ring tension-band metallic plate; SIJS: Sacroiliac joint screw; FE: Finite element; F2-CDH: The second generation Chinese Digitized Human; SIJCL: SI joint capsular ligaments; ASIL: Anterior sacroiliac ligaments; PSIL: Posterior sacroiliac ligaments; ISIL: Interosseous sacroiliac ligaments; SSL: Sacrospinous ligaments; STL: Sacrotuberous ligaments; SPL: Superior pubic ligaments; APL: Arcuate pubic ligaments; PL: Pectineal ligaments; IL: Inguinal ligaments.

## Competing interests

All authors seriously state that this new work has no conflict of interest relationships with other people or organizations.

## Authors’ contributions

HC participated in clinical cases control study, and LW participated in finite element model controlled trial. RZ participated in pre-processing of the intact and plate-fixation pelvis FE models, YaL participated in CT/MRI scan of F2-CDH and clinical data statistics, YanL participated in pre-processing of screw-fixation pelvis FE models, ZD participated in digital anatomy of the intact and fractured pelvis. HC and LW both participated in the study design and manuscript preparation. All authors read and approved the final manuscript.

## Pre-publication history

The pre-publication history for this paper can be accessed here:

http://www.biomedcentral.com/1471-2474/14/217/prepub
